# Identifying models of care to support residents in long-term care homes (LTCHs) both during and beyond COVID-19

**DOI:** 10.1371/journal.pone.0329255

**Published:** 2025-08-20

**Authors:** Lames Danok, Joanna Burke, Tanya MacDonald, Sidra Cheema, Sharon Straus, Christine Fahim

**Affiliations:** 1 Li Ka Shing Knowledge Institute of St. Michael’s Hospital, Toronto, Canada; 2 Healthcare Excellence Canada, Ottawa, Canada,; 3 Perley Health, Ottawa, Ontario, Canada; Hong Kong Shue Yan University, HONG KONG

## Abstract

Long-term care homes (LTCHs) implemented various models of care during the COVID-19 pandemic. The purpose of this study was to identify these models of care and provide suggestions on best practices that could be integrated into LTCHs in efforts to improve resident care. The project included a quantitative survey and semi-structured key informant interviews with LTCH managers across Canada. Our objectives were to 1) identify models of care that were used to support resident care in Canadian LTCHs during the COVID-19 pandemic and to describe their intervention components, processes of implementation, and perceived impact; 2) determine whether LTCHs planned to sustain models of care implemented during the COVID-19 pandemic. Our results show that the most frequently reported models of care were related to healthy food options, exercise, music and art programs, and planned social activities for residents. Five barriers were identified in relation to implementing these models of care, which included: lack of funding, resources, or staffing; staff not being familiar with/reluctant to use the model; lack of resident buy-in; fear of COVID-19; and pandemic regulations. Common facilitators to implementation were also identified and included: staff support; resident/family buy-in; funding, legislation and/or resources provided; familiarity with model prior to COVID-19; and collaboration with other LTCHs. LTCHs perceived the models to be effective and planned to sustain most implemented models. LTCH managers discussed the need for funding and legislation to improve LTCHs and support the implementation of promising models of care. This study provides insight into the models of care implemented during the pandemic crisis period in Canadian LTCHs, how effective they were perceived to be, and plans for sustainment beyond the pandemic period.

## Introduction

Long-term care homes (LTCHs) provide residential care for individuals who require assistance with their activities of daily living due to physical, cognitive, and/or medical conditions [1]. With the onset of COVID-19, a spotlight was placed on LTCHs due to high rates of virus transmissibility and increased risk of infection and death. The impact of COVID-19 on Canadian LTCHs was devastating. In Canada, during the first wave of COVID-19, 80% of COVID-19 deaths were among LTCH residents [2]. The incidence rate for death from COVID-19 was 13 times higher for residents in LTCHs compared to adults who were 69 years of age or older living in the community [3,4]. Staff working in LTCHs also faced greater inequities compared to other health care sectors [5]. For instance, staff in LTCHs were more likely to live in lower-income neighbourhoods, which had higher cases of COVID-19 compared to higher-income neighbourhoods [5–7].

COVID-19 outbreaks in LTCH were exacerbated by conditions such as poor ventilation, overcrowding, understaffing, and inadequate infection prevention and control measures [8]. Multiple measures were implemented to mitigate COVID-19 transmission including increased infection prevention and control measures, training for staff, and minimizing home visits [9]. Some LTCHs also made changes to their models of care. For example, in person family visits were shifted to virtual calls, introducing a new model of care for residents that relied on technology to maintain social connections [9].

The Ontario Long Term Care Staffing Study Advisory Group urged the Ontario Ministry to change the landscape of LTCHs, by suggesting various models of care that directly targeted both staff and residents [10]. One of the prevalent challenges faced by LTCHs was determining which models of care were effective and implementable admist the pandemic [1,11]. LTCH staff experience high levels of burnout and stress in their roles, and this was exacerbated during COVID-19 [12]. Limited staff capacity, coupled with low staff to resident ratios, limited funding, and access to service and supports further challenged LTCHs' ability to deliver care during the pandemic. [1,13].

Healthcare Excellence Canada (HEC) is working to improve the care of older adults across Canada, with a focus on the LTCH sector through partnerships with various organizations including Health Quality British Columbia, Manitoba Institute for Patient Safety (Shared Health), and the New Brunswick Association of Nursing Homes, as well as through collaboration with LTCH workforce members, leaders, residents and care partners [14]. In 2022, HEC sought to identify emerging models of care that were successfully implemented in LTCHs during the COVID-19 pandemic, with the intent of disseminating promising models through HEC’s Reimagining LTC program which aims to enable a healthy workforce to provide person-centred care [15]. HEC partnered with the Knowledge Translation Program at St. Michael's Hospital in Toronto, Ontario to conduct a survey and key informant interviews to 1) identify models of care that were used to support resident care in Canadian LTCHs during the COVID-19 pandemic and to describe their intervention components, processes of implementation, and perceived impact; 2) determine whether LTCHs planned to sustain models of care implemented during the COVID-19 pandemic. Our objectives were to understand the models of care, their implementation in LTCHs, and to determine promising practices to support LTCHs in the post pandemic period.

## Methods

This study received ethics approval from the St. Michael’s Hospital Research Ethics Board, REB #145-2013.

### Study design

The study was conducted in two phases. Phase I included a survey sent to LTCH managers across Canada, and Phase II included key informant interviews with LTCH managers. To inform the development of the survey and key informant interview guide, we generated a list of models of care commonly used in LTCHs. The list was informed by a literature review and input from LTCH and HEC staff [1,16–23]. We explored 12 models of care (Table 1) and invited participants to highlight additional models, particularly ones they felt were innovative or promising, for scaled implementation in LTCHs. In this study, we defined model of care as a way that a health service is designed or delivered [24].

**Table 1:** Models of care and descriptions.

Models of care	Descriptions
Task shifting [1,23]	Moving the care of some patient groups from health workers with higher levels of training to health workers with lower levels of training
Staff engaging families/essential care partners in resident care [17,22]	Incorporating family members and/or other essential care partners in supporting residents
Including the voice of residents in developing care models [17,21]	Incorporating residents’ thoughts, opinions, and feedback into their care
Staff education [16,18,20]	Providing staff with the appropriate knowledge and/or skills needed to better care for residents
Employee and Family Assistance Plans (EFAP) [18,19]	Services designed to assist employees who are experiencing personal and job-related problems that affect work performance, general health, and well-being
Peer support for staff [18,20]	Social supports for staff provided by colleagues at a similar job level
Healthy food options for residents [19]	Provision of nutritious and culturally appropriate foods for LTCH residents
Spiritual, worship services for residents [18,20,21]	Coordinating resident involvement with spiritual life, such as connecting residents with spiritual leaders
Mental health and wellness support and resources for staff [16,18,20]	Provision of therapies, wellness activities (e.g., guided meditation) for LTCH staff
Technology to connect residents with family and friends [22]	Use of technologies (e.g., iPads, Zoom) to connect LTCH residents with loved ones outside of LTCHs
Planned social activities for residents [18,21,22]	Organized internal events for LTCH residents to interact and spend time with one another and/or engage in LTCH community events
Exercise, music, or arts programs for residents [1,18,21]	Organized internal events for LTCH residents focused on physical activity, music, or art programs.

#### Phase I: Survey.

**Participants**

Participants completed an online screening survey to determine eligibility to participate. Those who identified as a manager of a Canadian LTCH and were able to complete the survey and/or interview in English were eligible to participate. We defined manager as anyone who oversaw the provision of service care in their LTCH and left this definition broad, given the variation of the role across homes.

**Recruitment**

We used passive and active recruitment to identify participants. We sent emails to our personal networks (e.g., St. Michael's Hospital *Wellness Hub Program* Newsletter), LTCH organizations, and LTCHs throughout Canada to facilitate recruitment. We also sent targeted invitation emails to 598 LTCHs in Canada; these LTCHs were identified from a list that was previously compiled to facilitate recruitment for a study titled Wellness Hub, that aimed to support Long Term Care and Retirement Homes in Ontario (mainly the Greater Toronto Area and Ottawa regions) to navigate the COVID-19 pandemic [25]. LTCHs were also identified manually using Google search functions for Canadian provinces and territories. Study advertisements were posted in online newsletters (i.e., through HEC program newsletters, and newsletters for LTCH specific organizations such as the Ontario Long Term Care Association (OLTCA)) and social media pages (i.e., OLTCA Facebook page). Aiming for representation from all provinces and territories, LTCH organizations were identified and contacted throughout Canada. We also invited managers who received our targeted emails to disseminate the survey to their professional networks. We did not ask participants which LTCH they worked at, thus multiple managers from the same LTCH could participate in the study. As of 2021, there were 2076 LTCHs in Canada, and we were able to contact 598 resulting in an approximately 29% response rate from LTCHs throughout Canada [26].

### Survey development and data collection

The online survey [27] was prepared by our research team with input from HEC and assessed for face validity; specifically, team members piloted the survey for clarity prior to dissemination. It included 6 core questions, with additional questions that appeared via branching logic, depending on participants' responses. For each of the 12 models of care, participants were asked: if they used the model in their LTCH during COVID-19, if they perceived the model of care to be effective at improving resident care; and (if implemented), whether they planned to sustain the model post-pandemic. Participants were also asked to list and describe any other models of care they implemented during the pandemic. We collected information on the type of LTCH in which the participant worked. We also collected optional demographic information about the participant, including their age, province they live in, languages they speak, their assigned sex at birth, gender, and ethnicity.

Participants were unable to change their answers once they had advanced through the survey. Cookies were tracked to prevent duplicate responses. Respondents were invited to participate in a follow-up interview to further discuss the models of care implemented in their LTCH, and their experiences with implementation. Participants who expressed interest received an external survey pop-up link that prompted them to enter their contact information in order to be contacted by a member of the research team. All survey questions were voluntary, and consent was collected prior to beginning the survey. All responses were anonymous, and we did not collect identifying information. Participants had the option of entering their email address upon survey completion to receive a $10 gift card. Participants' email addresses were stored separately from their survey data. Data was collected between August 23^rd^, 2022, to November 29^th^, 2022.

### Data analysis

Data were downloaded from the Qualtrics [27] server into Excel and summarized using descriptive statistics.

#### Phase II – Key informant interviews.

**Recruitment**. Email invitations were sent to survey respondents who expressed interest in participating in an interview. We aimed for demographic representativeness that reflected a range of intersecting identities (by gender, race, and province/territory). We also aimed to include managers from profit and not-for-profit LTCHs.

**Interview guide.** The interview guide listed the 12 models of care and asked respondents to identify the models they had implemented in their LTCH. For each model that participants implemented, we asked them to describe barriers and facilitators to implementation, perceived effectiveness of the care model, whether they planned to sustain the model post-pandemic, and whether they believed it should be integrated into LTCHs as a standard. Participants were invited to share whether other models of care (particularly those they perceived as innovative or promising) were implemented during the pandemic.

To further understand the factors impacting implementation, we probed for barriers and facilitators to implementation using the Consolidated Framework for Implementation Research (CFIR, for organisational factors) [28] and the Theoretical Domains Framework (TDF, for individual factors) [29]. We collected demographic information on participants’ LTCH setting, role, and responsibilities. The interview guide was prepared by the research team with input from HEC and piloted internally prior to use.

### Data collection

Participants had the option of scheduling a virtual (Zoom) or phone interview, at their convenience. Interviews were 30–60 minutes in length. Interviews were conducted between September 20^th^, 2022, to December 1^st^, 2022 by a Research Coordinator (LD) who did not hold any relationship with the study participants. Prior to interview commencement, participants were reminded about the purpose of the interview, given the terms of consent, and asked for permission to record their verbal consent. The participant was reminded that the interview would be anonymous to help limit socially desirable responses. Interviews were recorded and transcribed verbatim using NVivo Transcription Software. Transcribed interviews were uploaded for analysis into NVivo 12 qualitative software [30]. Participants who completed an interview were given a $50 gift card honorarium.

### Data analysis

We analyzed data in two stages: (1) framework analysis and (2) deductive coding to the CFIR [28] and TDF [29]. We followed the framework method as described by Gale et al. to analyze our data [31]. Analysis stages included (1) familiarization with data, (2) identifying the codes and themes across the interviews, (3) coding the data, (4) indexing codes and (5) charting codes [31]. A Research Coordinator (LD) and Research Assistant (SC) independently coded three transcripts that were randomly selected. Inter-rater reliability was determined by calculating kappa values. Coding discrepancies less than 0.60 were discussed and resolved via consensus. The remaining transcripts were single coded by the Research Assistant (SC). Barriers and facilitators were categorized to the CFIR [28] and TDF domains by one Research Coordinator (LD) [29].

## Results

### Phase I (survey)

#### Participant demographics.

A total of 435 individuals participated in the survey (Table 2). The majority of participants were from Ontario (38.2%) and British Columbia (23.2%). Approximately 50.9% of participants worked in a for-profit home, 45.9% worked in not-for-profit home, 1.9% worked in a home that was both not-for-profit and for-profit, 1.4% were not sure or preferred not to answer. With respect to gender, 50.6% of participants identified as men, 46.4% identified as women, 2.9% preferred not to answer. The participant sample was diverse with respect to race/ethnicity (see Table 2).

**Table 2:** Participant demographic information.

	Number of times selected (count and percentage of selection)
**Province**	
Ontario	163 (38.2%)
British Columbia	99 (23.2%)
Alberta	29 (6.8%)
Manitoba	28 (6.6%)
New Brunswick	25 (5.8%)
Nova Scotia	23 (5.4%)
Quebec	23 (5.4%)
Newfoundland and Labrador	18 (4.2%)
Saskatchewan	14 (3.2%)
Prince Edward Island	5 (1.2%)
**Funding of workplace**	
Privately funded (for-profit)	216 (50.9%)
Publicly Funded (not-for-profit)	195 (45.9%)
Both private and public	8 (1.9%)
Not sure/Prefer not to answer	6 (1.4%)
**Gender**	
Male	209 (50.6%)
Female	192 (46.4%)
Prefer not to answer	12 (2.9%)
**Age**	
18-44	117 (28%)
45-64	286 (69%)
65+	15 (3%)
**Ethnicity**	
White	159 (38%)
Black	96 (22.9%)
Other Racialized (Middle Eastern, East Asian, Southeast Asian, Latin)	72 (17.2%)
Prefer not to answer	92 (22%)

#### Models of care implemented in LTCHs during the COVID-19 pandemic.

The models of care most commonly implemented by participants during the pandemic included: healthy food options for residents (69%), exercise, music, or art programs for residents (54%), and planned social activities for residents (45%). Table 3 lists the prevalence of models of care implemented in our sample. Three additional models of care were mentioned by participants: the butterfly model, the clinical triad, and a social work navigator.

**Table 3:** Count and percentages of models of care selected by participants, their perceived effectiveness, and plans for sustainment.

Model of care implemented during COVID-19 pandemic	Number of times selected (count and percentage of selection)^1^	Do you believe this model of care has been effective in improving resident care? (count and percentage of sustainment)			Do you plan to sustain this model of care beyond COVID-19 pandemic? (count and percentage of sustainment)		
		Yes	No	Unsure	Yes	No	Unsure
Healthy food options for residents	300 (69%)	296 (99%)	0	2 (0.6%)	298 (100%)	0	0
Exercise, music or art programs for residents	237 (54%)	228 (97%)	1 (0.4%)	6 (3%)	229 (97%)	1 (0.4%)	5 (2%)
Planned social activities for residents	194 (45%)	191 (99%)	0	1 (0.5%)	191 (99%)	0	1 (0.5%)
Mental health and wellness support, and resources for staff	164 (38%)	148 (91%)	0	14 (9%)	156 (96%)	1 (0.6%)	5 (3%)
Technology to connect residents with family and friends	117 (27%)	102 (89%)	8 (7%)	5 (4%)	106 (92%)	1 (0.9%)	8 (7%)
Staff education	114 (26%)	109 (97%)	0	3 (3%)	108 (96%)	0	4 (3%)
Employee and Family Assistance Plans (EFAP)	113 (26%)	89 (79%)	5 (4%)	18 (16%)	102 (91%)	0	10 (9%)
Peer support	104 (24%)	93 (91%)	3 (3%)	6 (6%)	96 (94%)	2 (2%)	4 (4%)
Spiritual/worship services for residents	93 (21%)	82 (90%)	2 (2%)	8 (8%)	87(95%)	2 (2%)	3 (3%)
Staff engaging families/essential care partners in resident care^2^	288 (20%)	47 (96%)	1 (2%)	1 (2%)	47 (96%)	0	2 (4%)
Staff including the voice of residents in developing care models	81 (19%)	58 (75%)	9 (12%)	14 (18%)	64 (83%)	4 (5%)	9 (12%)
Task shifting for staff	58 (13%)	45 (76%)	5 (9%)	8 (14%)	37(64%)	4 (7%)	10 (29%)
Other: e.g., “Butterfly model,” “clinical triad to case manage residents,” “work as a team”	3 (0.7%)	3 (100%)	0	0	3 (100%)	0	0

^1^All questions were optional, participants who selected a model of care did not have to answer follow-up questions.

^2^Data on *Staff engaging families/essential care partners in resident care* were not collected until September 16, 2022, due to an error in survey production.

#### Perceived effectiveness of implemented models of care.

Participants generally perceived the implemented models of care to be very effective (Table 3). Almost all (99%) participants who implemented healthy food options for residents and planned social activities for residents perceived these models to be effective at improving resident care. Exercise, music, or art programs for residents was also perceived to be effective by most (97%) participants. Including the voices of residents in developing care models had the highest proportion of respondents (12%) who did not perceive the model as effective at improving resident care.

#### Plans to sustain models of care beyond the COVID-19 pandemic.

Participants generally had plans to sustain the implemented models of care beyond the pandemic crisis period (Table 3). All (100%) respondents planned to sustain healthy food options, while almost all (99%) and (95%) planned to sustain social activities for residents, and exercise, music, or art programs for residents, respectively.

Task shifting had the highest proportion of respondents (7%) who did not report plans for sustainability post-pandemic (Table 3).

### Phase II- key informant interviews

#### Participant demographics.

Thirty-seven (37) LTCH managers expressed interest in participating in the key informant interviews. Aiming for cross-country and demographic representation, email invitations were sent to 30 of those participants. Thirteen (13) of those who received the invite were still interested in participating, and 10 of those participated in an interview. The managers interviewed were from Ontario, Nova Scotia, New Brunswick, Saskatchewan, and Alberta. With respect to workplace funding, 5 managers worked for not-for-profit homes, and 5 worked in for-profit homes.

#### Identified models of care.

**Task shifting.** Participants who implemented task shifting (n = 5) did so to address the challenge of staff shortages. Participants perceived task shifting to improve residents' quality of life and care, as more staff were able to assist residents while maintaining their current levels of care. Funding and legislation assisted in hiring new staff to support the model.

**Staff engaging families/essential care partners in resident care.** Participants who engaged families/essential care partners in resident care (n = 6) perceived the model to improve resident care by improving advocacy. Some implemented this model following a Public Health authority directive, others implemented this model pre-pandemic as a strategy to engage and motivate staff, residents, and families.

**Staff including resident voices in developing models of care.** Participants who included the voice of residents in co-designing models of care (n = 5) did so due to an existing resident-centered focus. Some implemented the model due to their LTCH being bound by legislative standards to engage residents in their care. Participants perceived that residents felt heard, valued and respected with the implementation of this model, leading to improved resident care.

**Staff Education.** Participants who implemented staff education (n = 4) believed educating staff ensured safety and promoted staff confidence. They believed it also improved resident care, as staff were more confident in their roles.

**Employee and Family Assistance Plans (EFAP).** Participants who implemented EFAP (n = 3) wanted to provide staff with a safe and supportive environment during the COVID-19 pandemic. Participants believed EFAP was an effective first step in improving staff mental health and led to improved staff wellness, which was reflected in interactions with residents.

**Peer support for staff.** Participants who implemented peer support for staff (n = 2) did so to support staff wellness. They reported that staff were extremely appreciative of this model and that it provided staff with a sense of community. It was perceived to be effective in improving resident care, due to building strong rapport among colleagues. 

**Technology to connect residents with family and friends**. Participants who implemented technology to connect residents with family and friends (n = 8) recognized there was a significant disconnect between residents and their loved one during the pandemic, due to visiting restrictions, and that the technology was necessary to prevent residents from feeling isolated. Participants believed it improved residents’ psychological and emotional health.

**Mental health and wellness supports, and resources for staff.** Participants who implemented mental health and wellness supports for staff (n = 7) recognized the need to support staff who were experiencing mental health, wellness, and burnout challenges during the pandemic. This model was believed to support staff and build resilience, which allowed them to better care for residents.

**Spiritual, worship services for residents**. Participants who implemented spiritual or worship services for residents (n = 7) recognized the need for this service for residents, and the importance it held for them. Participants felt that incorporating a sense of shared community for residents was necessary and made them feel supported.  

**Planned social activities for residents**. Participants who implemented planned social activities for residents (n = 7) did so to reduce resident loneliness and deterioration of mental health. They perceived the incorporation of social activities to be beneficial for residents as it decreased loneliness and increased motivation for residents to interact with others.

**Exercise, music, or art programs for residents.** One participant implemented exercise, music, or art programs for residents, via programs that were already being offered in their home pre-pandemic. This participant perceived the residents to be grateful for and supportive of the initiative. It was believed to improve resident care, as it created more social opportunities for them.

**Healthy food options.** Participants who implemented healthy food options for residents (n = 2) noted that healthy food options were in place for residents pre-pandemic, but that their LTCHs put a greater emphasis on this model during COVID-19 to help keep residents healthy. Participants perceived that residents were happy with and appreciated the healthy food options.

**Post-pandemic use and integration into LTCH**. Managers said they would sustain the use of all implemented models of care post-pandemic. They also believed these models should be integrated into standard LTCH practices.

**Other models of care**. Three managers (n = 3) discussed other models of care they had implemented in their home. One manager described the implementation of the butterfly model which is a trademarked care model developed by UK-based *Meaningful Care Matters* to provide residents with more autonomy over their care [32]. This model was described by the participant as trying to remove the institutionalized feeling from LTCHs and giving residents the power to make their own decisions in their care and day to day routine.

One manager implemented the clinical triad, a case management approach to resident care. The manager described the model as including a registered nurse and two licensed practical nurses who case-manage 14 residents. These residents are provided continuous care that evaluates their progress, and the care team provides a consistent point of contact for the resident’s family.

One manager implemented a social work navigator who was assigned to ease the transition of the resident to LTCH. The social worker also provided continuity for the residents and their families.

**Barriers to implementation**. We identified five barriers to implementing the models of care. Barriers included: fear of COVID-19; lack of staff familiarity with model; lack of resident buy-in; lack of funding, resources, or staffing; and pandemic regulations.

Barriers that were most prevalent across all models of care were: lack of funding, resources, or staffing (n = 23), staff not being familiar with or reluctant to use model (n = 19), and lack of resident buy-in (n = 9).

See Table 4 for all models of care, and prevalent barriers found. See Table 5 for the definitions of identified barriers and illustrative quotes.

**Table 4:** Barriers to models of care.

Model of care	Barriers				
	Fear of COVID-19	Staff not familiar with or reluctant to use the model, or have differing perceptions about the model impact/benefit	Lack of resident buy-in (i.e., residents are not able to/ do not support implementation of model)	Lack of funding, resources, or staffing	Alignment with pandemic regulations (i.e., social gatherings prohibited due to COVID-19)
Task shifting	X (n = 1)	X (n = 3)			
Staff engaging families/essential care partners in resident care		X (n = 4)		X (n = 1)	X (n = 1)
Staff including the voices of residents in developing care models		X (n = 2)	X (n = 3)		
Staff education		X (n = 1)		X (n = 2)	
Employee and family assistance plans (EFAP)		X (n = 3)			
Peer support for staff				X (n = 1)	
Healthy food options for residents				X (n = 2)	
Spiritual, worship services for residents		X (n = 2)		X (n = 3)	
Mental health, wellness support and resources for staff		X (n = 2)		X (n = 6)	
Technology to connect residents with family and friends		X (n = 2)	X (n = 5)	X (n = 7)	
Planned social activities for residents	X (n = 2)		X (n = 1)	X (n = 1)	X (n = 5)
Exercise, music, or art programs for residents					X (n = 2)

**Table 5:** Definition of barriers and illustrative quotes.

Barrier	Barrier, as informed by the consolidated framework for implementation research [28] and theoretical domains framework [29]	Description of barrier	Illustrative quotes
Fear of COVID-19	Emotion (fear) (19)	Fear of COVID-19 by staff, residents, families of residents	“People at the beginning of the pandemic were so fearful of even being in a building or in a collective type of setting.” Participant 102 “I think there was still fear largely on the behalf of the staff in bringing the residents back together because they didn’t want to cause an outbreak or cause somebody to get sick.” Participant 108
Staff not familiar with model; are reluctant to use model; or have differing perceptions about the model impact/benefit	Knowledge and skills; Beliefs about consequences (19)	Staff not familiar with the model, or have differing views/beliefs about model	“I think this is a generation thing too. Some of my staff are not familiar with you know, turning on phones, computers, so we have to go back to the basics.” Participant 105 “The first challenge was the recognition that we had a very diverse group of individuals, all of whom come with different background and different educational preparation.” Participant 103
Lack of resident buy-in	Lack of model fit with the residents’ personal circumstances and health status (18)	Lack of appropriate strategies to engage residents with communication and cognitive impairment	“Some of the residents, you know they can’t communicate effectively, some of them have a cognition issue.” Participant 106 “The cognitive level wasn’t there to understand that they were calling from home, but they could see a picture through the phone. That was alien to some of our residents.” Participant 110
Lack of funding, resources, or staffing	Financing, relative priority, and staff skill (18)	LTCHs not provided with adequate funding or resources to assistant in implementation	“Resources was the biggest one…getting the proper technology to implement our goals, eventually we came up with, were successful getting some iPads but they were very few and far between.” Participant 104 “We don’t have a psychologist or psychiatrist, so we don’t have the area of expertise.” Participant 101
Alignment with pandemic regulations	Policies and laws (i.e., pandemic regulations) (18)	Government or LTCH regulations/policies limit aspects of implementation	“What our challenge was, was to figure out how do we increase the level of activity and the level of opportunities to participate in activities while at the same time trying to do all of that in the middle of cohorting residents because of the pandemic.” Participant 101

#### Facilitators to implementation.

We identified six facilitators to implementing the models of care. Facilitators included familiarity with the model before COVID-19; collaboration between staff/teamwork; resident and family buy-in; funding, legistration and/or resources; pre-existing model supported implementation of new model; and collaboration with other LTCHs.

The facilitators that were most commonly described across all models of care were: collaboration between staff/teamwork (n = 24), resident/family buy-in (n = 21), and funding, legislation and/or resources provided (n = 13). Table 6 describes the facilitators; Table 7 provides definitions of the facilitators and illustrative quotes.

**Table 6:** Facilitators to models of care.

Model of care	Facilitators					
	Familiarity with model before COVID-19	Collaboration between staff/ teamwork/support	Resident/family buy-in	Funding, legislation, and/or resources provided	Pre-existing care model supports implementation	Collaboration with other LTCH’s
Task shifting		X (n = 3)	X (n = 1)	X (n = 1)	X (n = 1)	X (n = 1)
Staff engaging families/essential care partners in resident care		X (n = 1)	X (n = 4)		X (n = 1)	X (n = 1)
Staff including the voices of residents in developing care models		X (n = 2)	X (n = 2)		X (n = 2	
Staff education		X (n = 3)		X (n = 1)		
Employee and family assistance plans (EFAP)		X (n = 2)				
Peer support for staff		X (n = 2)				
Healthy food options for residents				X (n = 1)	X (n = 1)	
Spiritual, worship services for residents	X (n = 1)		X (n = 2)			
Mental health, wellness support and resources for staff	X (n = 1)	X (n = 5)		X (n = 2)		X (n = 2)
Technology to connect residents with family and friends	X (n = 2)	X (n = 4	X (n = 5)	X (n = 6)		X (n = 1)
Planned social activities for residents		X (n = 2)	X (n = 5)	X (n = 2)		
Exercise, music, or art programs for residents	X (n = 2)		X (n = 2)			

**Table 7:** Definition of facilitators and illustrative quotes.

Facilitator	Facilitator, as informed by the consolidated framework for implementation research (CFIR) [28] and theoretical domains framework (TDF) [29]	Description	Illustrative quote
Familiarity with model before COVID-19	Knowledge and skills (19)	Staff/ homes were familiar with components/ concept of model before COVID-19	“The residents are, you know they’re already using this, because some of the family members here are not necessarily in Ontario, they’re actually in other provinces.” Participant 103 “The technology was all either in place or being put in place before the pandemic, and in this case mostly that on the part of external parties, the churches.” Participant 107
Collaboration between staff/ teamwork	Relational connections and communication (18)	Staff is collaborative and communicates effectively/ is supportive to implement model of care	“Especially in the beginning. I think it’s getting old now but, in the beginning, it was amazing how the teams came together.” Participant 105 “The staff was so receptive. Knowing this is long-term care environment, and the need to have the proper information to come to work every day and feel at ease.” Participant 104
Resident/Family buy in	Characteristics of the model of care, as perceived by residents (i.e., innovation relative advantage) (18)	Residents or families of residents are supportive of model of care (in comparison to existing or other models of care)	“It made it easy that we had the support and the time to meet with families, have families who were engaged and positive and wanting to do what we at the time knew with the information that was safest for the residents.” Participant 102 “There was enthusiasm from some of the residents and families and that’s a major driving factor for me. If they’re excited about something even if it’s as simple as seeing a family member that’s very motivational.” Participant 107
Funding, legislation, and/or resources provided	Financing, available resources, policies & laws (18)	The LTCHs are provided with adequate funding or resources, or legislation to facilitate implementation of model of care	“I think the Ministry funding helped out a lot. We were able to get a lot more technology and staff into the building to set that up.” Participant 106 “We had additional funding from the government to have a long-term care assistant, and those individuals were very instrumental in the early stages, when families were not able to come in." Participant 109
Pre-existing care model supports implementation	Compatibility (18)	LTCH's existing model(s) facilitated implementation of the new model of care	“I would say it was existing as part of the structure of the care that is supposed to be delivered for the resident and it’s also part of the program itself.” Participant 102 “The other thing that made it was there’s this sort of an internal focus on resident-centered care, and this just became part of also operationalizing resident-centered care.” Participant 104
Collaboration with other LTCHs	Partnerships & connections (18)	Communication between other LTCHs assists in implementation of model of care	“We felt supported in the sense that another home had shared their experience so all the work they had done they shared with us, so we didn’t have to start from scratch.” Participant 109 “We as a LTCH we have our community and when we talk to each other we can now look at what is happening in your home. What are you doing in this sort of home? We kind of share information, what’s good, what your challenges are, and it helps us to build on that.” Participant 103

#### Opportunities for system improvements.

Dedicating funding to support implementation of models of care in LTCH and outlining policies/legislation to require or standardize these models were suggestions provided by LTCH managers.

Lack of funding was a significant barrier to implementation, cited by all interview participants (n = 10). Funding was required to better support staff, secure technology, and provide healthy food options. Participants cited the need for increased resources and funding to allow for consistent programs across LTCHs. Funding was also required for consultants to provide professional support. Participants (n = 8) noted that this funding was a direct facilitator to implementing models.

Participants also noted the impact of legislation on implementing models of care consistently across LTCHs. Notably, one participant cited the importance of a policy that would empower employers to provide mental health self-care resources to their employees. Other suggestions for legislation included checklists for regulators/licensing, incorporation of resident feedback, infection prevention and control training and assistance, and staff education. Participants highlighted the positive impact of having these legislations/policies in place.

## Discussion

We aimed to understand the models of care being implemented in LTCHs during and following the COVID-19 pandemic, in order to inform best practices for sustainable LTCH models. Our study found that the most frequently reported models of care implemented in LTCHs during the pandemic crisis period were related to healthy food options, exercise, music, and art programs, and planned social activities for residents. These models were perceived by our participants to improve resident care and LTCH managers intended to sustain them post-pandemic. 

We identified barriers to implementation of these models of care including lack of funding, resources, or staffing; staff not being familiar with/reluctant to use the model; lack of resident buy-in; fear of COVID-19; and pandemic regulations. These barriers have been reported in the LTCH literature prior to COVID-19, demonstrating the sustained challenges exacerbated by the pandemic and the need for urgent restructuring to support both residents and staff [33–39]. Interestingly, our study found that most of the models of care that we assessed were perceived to be sustainable, even in face of these challenges. We also observed some variation by home, likely due to Canada's system of LTCH governance under provincial and territorial jurisdictions, which results in variation in resources, policies and the models of care adopted.[17,18,40] For example, some managers were able to secure funding to purchase tablets and facilitate virtual family visits, while others cited funding shortages that delayed implementation of such models.

Facilitators to implementation included staff and a LTCH context that supported the implementation of the models of care; resident/family buy-in; funding, legislation and/or resources provided; familiarity with model prior to COVID-19; and collaboration with other LTCHs. Notably, frequently exhibited models of care align with the recently released National Long-Term Care Services Standard developed by the Health Standards Organization (HSO), which offers guidance on the delivery of high-quality, evidence-informed, person-centered care and may support the standardization of care models across the country. [40] Similar guidance has also been established at the provincial level. These models also point to the key themes of person-centered care approaches and support for the workforce as emerging post-pandemic priorities for LTCHs [13].

In this post-pandemic period, there is also an emphasis on developing care standards [35] that also consider the needs of LTCH staff, who continue to experience significant burnout, moral injury and impact on mental health that peaked during COVID-19 [12]. Ensuring resilience of LTCH staff and empowering LTCH managers to facilitate strong communications and morale can have a direct impact on resident care and facilitate LTCH ability to sustain models of care [12]. Moving forward in the post-pandemic period, maintaining the increased focus on mental health and care strategies for staff and residents is crucial [41]. One recommended strategy has been to re-evaluate Employee Assistance Programs to ensure workers are able to access and utilize these services to their benefit, and to recognize the intersecting challenges often experienced by LTCH employees [12,42].

There are some limitations to this study. Participants were limited to those who could complete a questionnaire and/or key informant interview in English. This leads to the exclusion of perceptions and experiences of individuals who do not speak English. The sample size for our key informant interviews was small, with only 10 interviews included in the analysis, thus the results from the interviews are limited and may not capture the models of care and perspectives from managers of LTCHs across Canada. This study also only includes participants from the Canadian provinces and lacks representation from the territories, thus the results may not be applicable to that context. Future research should include more representation across Canada (including non-English speakers, and participants from the territories) and outside of Canada, to inform generalizability of these models. This study is also only focused on perceived impact of the models of care as reported by the participants and does not include a quantitative analysis of actual impact. During our interviews, funding was consistently cited as both a barrier and facilitator, however, we did not probe as to whether funding provided to homes at time of the interview was temporary or sustained funding post-pandemic. We also probed on certain models of care, which may have posed a leading bias. Due to timing and funding limitations, we were unable to pilot the survey, thus we were unable to ensure all questions within the survey captured all necessary models of care or were clearly understandable from the perspective of a LTCH manager. Lastly, participants identified the models of care that they wanted to discuss in the key informant interviews during the allocated time, and as such due to participant choice and time constraints, not all models were discussed in depth.

## Conclusion

This study used a survey and key informant interviews to explore models of care that were implemented in LTCHs across Canada during the COVID-19 pandemic and provided suggestions on best practices that could be integrated into LTCHs to potentially improve resident care. Participants discussed various models of care, their perceived effectives, barriers and facilitators to implementation, and plans for sustainment. Participants emphasized the need for increases in funding and legislation to facilitate successful implementation of these models.

The pandemic forced LTCHs to rapidly adapt new care models and optimize existing care models to limit the spread of COVID-19. The care models adopted can lead to large structural and organizational changes to improve the LTCH beyond the post-pandemic crisis period and impact care practices moving forward.

## Supporting information

S1 File Infographic summarizing key findings and recommendations from the study on improving long-term care services in Canada. (PDF)
