# Supplementary material for: Identifying models of care to support residents in long-term care homes (LTCHs) both during and beyond COVID-19
Source: PLoS One. 2025 Aug 20;20(8):e0329255. doi: 10.1371/journal.pone.0329255 (PMC12367117; doi:10.1371/journal.pone.0329255)
Supplement: S1 File — (PDF) [file pone.0329255.s001.pdf]

Models of care in long-term care homes during the COVID-19 pandemic

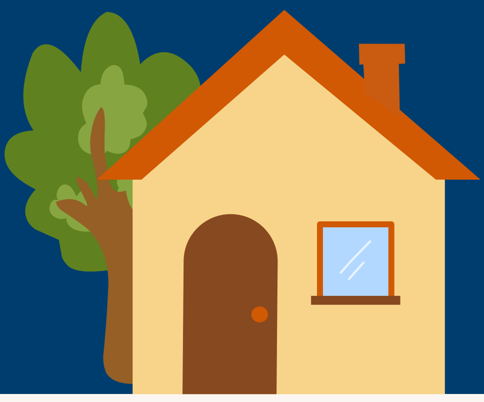

Purpose

Identify these models of care and provide suggestions on best practices that could be integrated into LTCHs to potentially improve resident care.

Objectives

1.
- Identify models of care that were used to support resident care in Canadian LTCHs during the COVID-19 pandemic*
  - Describe their intervention components, processes of implementation, and perceived impact

2.
- Determine whether LTCHs planned to sustain models of care implemented during the COVID-19 pandemic.

Results

Top reported models of care

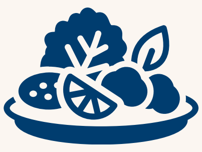

Healthy food

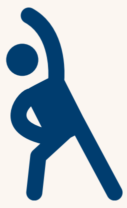

Exercise

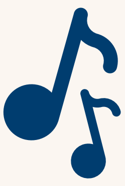

Music

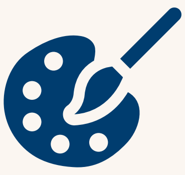

Art programs

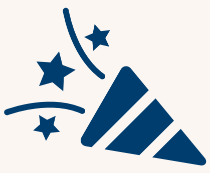

Social activities

Barriers

- lack of funding, resources, or staffing
- staff not being familiar with/reluctant to use the model
- lack of resident buy in
- fear of COVID-19
- pandemic regulations

facilitators

- funding
- staff support
- familiarity with model prior to COVID-19
- resident/family buy in
- collaboration with other LTCH's
- legislation and/or resources provided

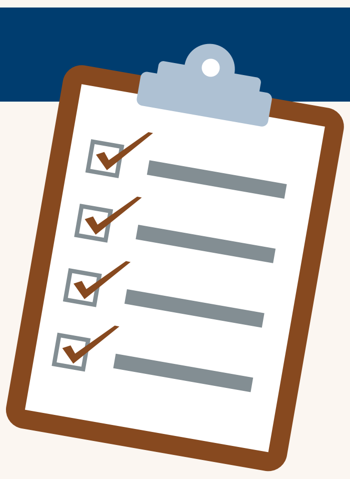

This study found that LTCH's planned to sustain most models of care post pandemic. LTCHs also perceived these models to be effective and believed they should be integrated into LTC as a whole. Managers also discussed the need for policy makers to focus on funding and legislation to improve LTC and support the implementation of promising, effective models of care.

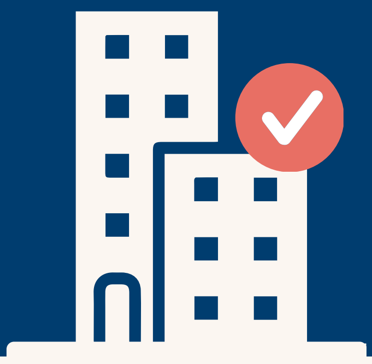

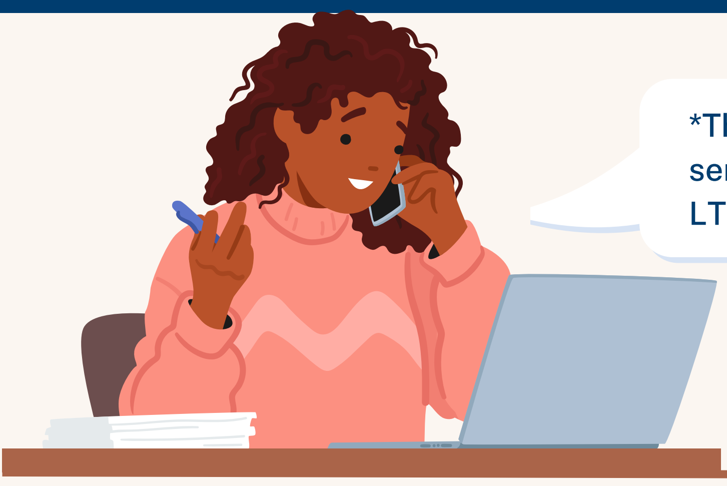

*The project included a quantitative survey and semi-structured key informant interviews with LTCH managers across Canada.
